# Mental Health Apps in Psychiatric Treatment: A Patient Perspective on Real World Technology Usage

**DOI:** 10.2196/12292

**Published:** 2019-04-22

**Authors:** Emil Chiauzzi, Amy Newell

**Affiliations:** 1 PatientsLikeMe, Inc Cambridge, MA United States; 2 Wistia Cambridge, MA United States

**Keywords:** bipolar disorder, mobile phone, mHealth, mental disorders, mental health, mobile applications

## Abstract

For many people who use mobile apps, the primary motivations are entertainment, news, gaming, social connections, or productivity. For those experiencing health problems, particularly those with chronic conditions such as psychiatric disorders, the stakes are much higher. The digital tools that they select may be the difference between improvement and decompensation or even life and death. Although there has been a wide expansion of mental health apps with promise as well as hype, the current means of researching, evaluating, and deploying effective tools have been problematic. As a means of gaining a perspective that moves beyond usability testing, surveys, and app ratings, the primary objective of this patient perspective is to question the *killer app* and condition-specific mentality of current mental health app development. We do this by reviewing the current mobile mental health app literature, identifying ways in which psychiatric patients use apps in their lives, and then exploring how these issues are experienced by a software engineer who has struggled with her bipolar disorder for many years. Her *lived experience* combined with a technology perspective offers potential avenues for using technology productively in psychiatric treatment. We believe that this responds to *JMIR Publications’* call for patient perspective papers and provides encouragement for patients to share their views on mental health and technology.

## Introduction

### Background

Mental illness is a common condition in the United States, as 44.7 million adults have been diagnosed with these disorders in the past year [1]. These statistics include 10.4 million Americans with serious mental illnesses such as major depression, anxiety disorders, and schizophrenia, but only 6.7 million have received treatment in the past year. To address patient difficulties accessing traditional mental health services, digital health tools offer the potential to scale treatment [2]. There are now over 300,000 mobile health apps available worldwide, with mental health accounting for the largest proportion of the rapidly growing disease-specific app market segment [3]. Owing to the broad use of mobile phones by people with mental health problems [4], it is not surprising that many patients have expressed an interest in exploring the use of these apps as part of their treatment efforts. Accordingly, these trends have driven an upsurge in clinical app research in anxiety, major depression, post-traumatic stress disorder, bipolar disorder, schizophrenia, and other mental health disorders [5-10].

### Challenges in Applying the Killer App Mentality to Mental Health Treatment

#### Finding the Features That Patients and Clinicians Want

In many cases, developers are seeking to design and market *killer apps* defined as follows:

A new software application used to attract consumers and motivate new hardware device purchases. Often innovative and cutting edge, killer apps are known for creating a large following. Over time, killer apps become an essential factor related to hardware or device purchases.

11

The *killer app* concept suggests that a single app can meet the needs of the target audience. However, this may be a questionable assumption with patients who have mental disorders.

From the perspectives of patients and clinicians, market share and profits are less of a concern than usability and successful treatment outcomes. What would a perfect *killer app* look like and what do patients want? Patients may not use the term *killer app* but, in general, they report an interest in using their mobile phones to track their mental health [12]. Symptom-monitoring is generally considered helpful and supportive by people with mental health problems [13]. Unfortunately, mental health app developers often *overengineer* their solutions rather than capitalize on the basic popular functions such as texting communication and medication adherence [14,15]. Patients seem less focused on elaborate features and more on optimizing their immediate goals (eg, daily functioning) and may require multiple apps to achieve these goals [16]. Lifestyle factors such as managing fitness, nutrition, lifestyle and stress, and diet and nutrition are critical in mental health [13] and are the most common reasons why people with chronic diseases download health apps [17].

Despite these proposed standards and patient needs, mental health apps often lack key features and suffer from inconsistent patient engagement. Critical features such as tailored feedback or recommendations for response to emergency or high-risk situations or features that support lasting behavior change are generally lacking [18]. In terms of engagement, 23% of users abandon mobile apps after 1 use [19], and retention is highly variable even within controlled studies among mental health users [2]. Adherence is an issue, as downloaded mood apps are used as intended for no more than 2 weeks [20]. These challenges may be magnified with older psychiatric patients who have serious and persistent disorders [21]. On the basis of a lack of highly controlled, longer-term outcomes in available research, there is no strong case for recommending any particular mental health mobile app [22].

In addition, despite general agreement on desirable app features and characteristics, the determination of quality seems to be in the eye of the beholder when evaluating individual apps. Available evidence indicates that ratings of effectiveness, ease of use, and performance have poor inter-rater reliability among professional stakeholders (clinicians, researchers, and technology experts) [23]. Among patients, mental health apps are more widely available in app stores, so users are generally left to evaluate their app choices based on user ratings. This is a questionable strategy, as the level of comprehensiveness of information and adherence to best-practice guidelines do not correlate with average user mental health app ratings [9]. Moreover, 1 study of almost 1000 depression apps found that only 35% were clinically relevant and only about 3% of these reported clinical effectiveness [8], suggesting that users should be careful about interpreting 5-star ratings. Taken together, these findings suggest serious challenges in defining and creating *killer* apps from a clinical quality perspective.

#### Determining What Works

Despite suggestions that mental health apps for certain conditions (eg, depression or anxiety) may be worthy of consideration in clinical guidelines [3], there are a variety of clinical and research challenges that make it difficult for clinicians and patients to identify tools that can supplement treatment efforts. First, a variety of studies and meta-analyses have demonstrated promising early outcomes, but findings are limited by a lack of appropriate control groups, short follow-ups, poor definition of content, lack of clinically valid mental health information, and poor usability [5-8,18,24]. Mobile health app development is driven more by commercial than scientific motivations [25]. Researchers tend to focus more on safety and efficacy, whereas commercial entities focus more on user engagement [26]. In 1 systematic review of 3000 mental health apps available on the Android, Apple, and Microsoft platforms, only 8 were evidence-based [24].

#### Deciding How to Integrate Apps Into Clinical Sessions

Mobile health apps for health care are generally not distributed through health care providers or settings [27], likely owing to a variety of barriers. Furthermore, 1 survey found that 77% of clinicians refer patients to informational websites but only 19% suggest the use of mobile apps to their patients to enhance clinical communication [28]. For clinical app usage to take hold, clinicians need to be more willing to collect patient data, require training in interpreting data outputs [29], and may have hesitance about billable time, therapeutic boundaries, privacy, and liability [30]. Attitudinal issues may be prominent, as clinicians may adopt paternalistic attitudes and have doubts about the validity of clinical information in digital tools [31]. There has also been concern about *digital transference* and the potential risks of digital technology to the provider-patient relationship [32]. Among patients, technology-based therapies may not be viewed as a replacement for live support [33]. There are indications that mobile mental health apps work better when paired with live support [34-36], but it is not clear if these apps provide added value to traditional face-to-face psychotherapy or treatment-as-usual [22]. For clinicians to accept mobile app solutions, apps need to be optimized to address technology competency, available resources, reimbursement, regulatory issues, and organizational infrastructure [37]. Without such optimizations, clinicians may not be well positioned to recommend mental health apps.

#### Taking Safety and Privacy Precautions

Finally, there are significant deficiencies in many apps with regard to potential harms that might result from inadequate safety or privacy protections, clinically questionable content, and potential stigma [38,39]. Many people are suffering enormously, lack even basic access to psychiatric care, may feel disrespected by the doctors they do get to see, and are not receiving adequate symptom relief. Under those circumstances, it is not surprising that people actively seek to diagnose and treat themselves. However, based on the questionable content in many apps, these individuals may not attain symptom relief or potentially experience harmful effects. Among those who are experiencing mood disruptions within the normal range, mental health apps may promote medicalization of symptoms [40]. In addition, users are seldom aware of the extent of data collected, the potential for these data to be sold to third parties, and the ways in which the data are used to drive risk calculations and personal profiling [38]. Torous et al [41] sum up the current state of affairs by stating that the “growing consensus is that most commercially available apps are not evidence-based and some are even dangerous.”

### Can Single Disease-Specific Apps Do it All?

In consideration of patient usage of mental health apps (and apps in general), previous research has highlighted the importance of usability and engagement [18]. Formal usability testing and iterative design with individual users are the preferred ways to optimize software performance [42]. However, we would like to expand this discussion to patterns of app usage as part of the lived experience of mental health patients. Given the profound effect that psychiatric disorders have on the quality of life [43], patients need to find solutions for problems on many fronts, so reliance on a single mental health app may not be realistic.

Qualitative studies suggest that people with chronic conditions do not rely solely (or even primarily) on disease-specific apps for management of their health [4,16,17,44]. Despite a high level of expressed interest, only 10% of psychiatric patients use mental health apps [45]. Some patients who are managing diabetes, depression, weight, and sleep issues may rely on a fitness app without using a disease-specific app [44]. Patients may use a mixture of apps focused on their menstrual cycles, blood pressure, calorie and weight management, pain management, fitness, or sleep [44]. Mental health patients may utilize multiple, widely available, and popular health apps to cope with their symptoms and can be highly creative, for example, coloring, brainteaser, and day planner apps [16], perhaps to manage cognitive symptoms. People with schizophrenia responding to a National Association of Mental Illness survey reported that their most common use of technology as a coping tool was to listen to music or audio files to block or manage auditory hallucinations [46]. Indeed, the distinctions between mental health and other apps may be more important to researchers and clinicians than patients [4].

On the basis of the above challenges, Hatch et al [36] have concluded that *the killer app approach is not the right mindset*. As a result, the primary purpose of this paper is to consider how individuals with mental health disorders can capitalize on more flexible and individualized app strategies to address self-management of their conditions. In our view, such considerations should be patient-centered, that is, what patients want and need, how can apps be best integrated into mental health treatment, and pragmatic, patient-centered technology capabilities that can be mobilized to address patient needs in more immediate ways. The issues above highlight a convergence between what is found in the literature and lived experience in managing psychiatric symptoms. We question the practicality of relying on single, condition-specific mobile mental health apps instead of focusing more flexibly on the diverse needs of mental health patients. This may involve the use of multiple apps and/or the use of condition-specific apps.

In response to *JMIR Publications’* encouragement for patients to submit their perspectives on mental health and technology, we offer Amy’s experiences with the challenges described above, as well as her real-world recommendations. The latter are based both on her history of psychiatric treatment and her background in software development. Our goal is not to suggest that one individual can speak for all but to offer potential hypotheses and creative solutions that may drive the conversation toward a more flexible technology-based approach in mental health clinical care.

### Amy’s Perspective on the Role of Apps in Mental Health Treatment

I am a 43-year-old woman who has suffered from bipolar disorder since the age of 9. I have treatment-resistant rapid-cycling type 2 bipolar disorder and spend about 80% of my days mildly to severely depressed. I frequently struggle with challenging symptoms like panic attacks, delusional hypochondria, suicidal ideation, irritability, despair, anhedonia, and exhaustion. I have self-harmed, and I have self-medicated. I understand that my disease is chronic, it is debilitating, and there is no cure for it. I have tried multiple modes of treatment over decades, including over a dozen medications (lithium, Depakote, multiple antipsychotics and antidepressants, multiple benzodiazepines), electroconvulsive therapy, and ketamine infusion, as well as multiple modes of therapy (dialectical behavior therapy, cognitive behavior therapy, acceptance and commitment therapy, mindfulness, etc) and alternative treatments (exercise, fish oil, meditation, reiki, etc).

Despite my struggles, I have managed to build and maintain a life that I value. I am a highly engaged patient, and I know many other people who are trying to do the same. I have spent decades working in the software industry, including 8 years as a software engineer at PatientsLikeMe, a community website for patients. This combination of experiences has led me to adopt a problem-solving attitude toward living with serious mental illness. In my view, the question is not what will fix or cure me, but what tips, tricks, apps, processes, books, resources, clinicians, and caregivers can help me live as well as possible with my disease.

On the basis of my own experience as a patient with bipolar disorder and as a software engineer, I would like to move the conversation away from what needs to be developed that will somehow solve the crisis of modern mental health care. How can clinicians who would like to use digital technology now to help their patients with mental illness live better today do so? What kinds of apps have I and other people with mental illness found helpful, and how can clinicians integrate those apps into their care? What are some general recommendations for evaluating apps and making use of them?

**Table 1:** Suggestions for app selection.

Challenge, key considerations		Strategy	Potential tools
**Finding the features that patients want**			
	Mindfulness	General wellness behavior; Use push notifications	Headspace [48]
	Help with daily life	Calendar, *to do*, banking, budgeting, laundry, and food delivery apps	Productive [49]; Bullet Journaling; Ridesharing apps such as Uber and Lyft
	Informed choices about medications	Get information about the use and effects of medications, balance difficult choices about side effects, integrate medications into a daily routine	Stahl’s psychopharmacology [50]; Iodine [51]
	Distress tolerance	Music to motivate physical activity, Facebook photo groups, and games for distraction	Apple Watch [52] electrocardiogram function
	Physical activity	Exercise as an evidence-based tool for symptom management	Yoga apps; Apps that book exercise classes; Activity trackers: Fitbit, Apple Watch [52]
**Determining what works**			
	Experiment with different apps	Offer trial periods and then are 1-time purchases or are sold on a freemium model	Web-based technology reviews, for example, New York Times and Wirecutter [53]
**Deciding how to integrate apps into clinical sessions**			
	Secure communications with clinicians	Crisis help and status updates	Signal [54]
	Tracking	Mood tracking; Important for clinician to explain evidence base for tracking recommendations; Clinicians should show a commitment to tracking by reviewing data regularly in sessions	Daylio [55]
**Taking safety and privacy precautions**			
	Crisis help	Keep key support phone numbers readily accessible as favorites	Crisis hotlines, numbers for providers, and local Samaritans
	Privacy and security	Understand personal tolerance for data sharing; Privacy and security are real concerns and cannot be guaranteed	Use secure messaging tools with providers

From the standpoint of a patient, I think it is important that clinicians avoid *prescribing* apps. It is much better to start from what problems patients are trying to solve, not from clinical ideas of what apps they should use and why. I have found it more helpful when I am engaged in treatment choices as problem-solving rather than being directed in what to do. The truth is people do not do things just because their doctors tell them to. They need to see the benefit of what they do.

Clinicians may think a patient needs to track their medication adherence with a particular tracking app, but both parties should agree that this is a priority. If not, patients are unlikely to do so. The problem of engagement and retention for mental health apps is no different than that for other kinds of apps; it is enormously difficult to create habits in other people. Apps with high retention (*adherence*) either have push notifications and *dopamine hits* going for them (social apps) or they are providing features and services that users find valuable [47].

The following sections review my personal experiences in the use of technology tools in my treatment. Table 1 summarizes the preferences and strategies that work for me or others whom I know but which may or may not work for others.

#### Finding the Features That Patients Want

I would like to see clinicians expand their understanding of what kind of apps might improve outcomes for the mentally ill. In general, I prefer apps that have only a few features to those that try to do too many things at once. In my experience, more features reduce usability, which reduces the likelihood of using the app. Using multiple apps for different purposes is not a problem for me if each app works well. In my experience, there are several broad areas where such apps can help the mentally ill live well.

##### Mindfulness

There is solid evidence that practicing mindfulness is helpful for a wide variety of symptoms, and practicing mindfulness is increasingly viewed as a general wellness behavior that improves psychological health [56]. As a result, there are multiple good apps out there to support mindfulness practice, including subscription-based, single-time purchase and free apps. I have practiced mindfulness as a component of my own wellness plan for 15 years, and I find that using an app is helpful.

What do I look for? I prefer apps that are marketed to the general consumer, so I do not feel stigmatized as a patient for using it—apps for people rather than patients. Push notifications remind me to meditate at a set time every day and offer me the opportunity to practice mindfulness in other moments throughout the day. My app also tracks the total minutes I have meditated since I started using it, which is motivating. Variety is important. For example, I use my meditation app to help me sleep, deal with anxiety, be mindful while doing chores or walking to work, or calm down in an intense moment. I also find it useful to get data about my use of the app. For example, Headspace (the app that I use) emailed me to let me know that I had meditated over 200 days in the last year. This made me feel good about my efforts to take better care of myself even if it is not a perfect daily habit. Many of the features I mention here are available in other mindfulness apps that are widely available, high-quality, and pleasant to use. All of these qualities may increase retention (ie, what clinicians might call *adherence*). In my view, mindfulness apps are the closest thing we have to *killer apps* for mental health because of the evidence for the effectiveness of mindfulness in reducing the suffering associated with mental illness [57].

##### Help With Daily Life

It is important for clinicians to explore with their patients what kinds of problems they are facing living their day-to-day lives and whether there are apps that can assist them. Many of the mentally ill suffer from problems with organization and executive functioning; difficulties completing daily tasks like getting around, managing their money, and engaging in basic habits of self-care [16]. As a result, clinicians and patients should cast a wide net for what might be helpful. For example, apps that make public transit easier to use may also help people get out and about more. Calendar, *to do*, banking, budgeting, laundry, and food delivery apps may all help patients solve daily living problems and allow them to live richer lives. I have an app called Productive (Table 1) that allows me to create recurring *to-dos*. Swiping away each *to-do* when I brush my teeth or take a shower gives me a feeling of pleasure and a sense of accomplishment. I often panic on public transit, am too tired to walk places, and too medicated or manic to feel that I can safely drive. Ridesharing apps like Uber and Lyft help me get where I want to go so that I can live my life without having to overcome as much anxiety or inertia. When I have trouble focusing, making use of an app that blocks my use of other apps helps me get more done. Again, there are multiple apps out there, of varying degrees of quality, and patients should be encouraged to explore and find what works for them. Most of them will not stick. That is fine. Some will.

##### Help Making Informed Choices About Medication and Then Taking the Medication as Prescribed

I understand that medication adherence is a huge issue for clinicians. In my experience, every day brings very challenging considerations and decisions about taking medications. Apps can help patients both gather medication information and manage their medication regimens. There are significant difficulties around getting information about the use and effects of medications, around balancing difficult choices about side effects, and around merely integrating medications into a daily routine. In my own experience, clinicians are frequently short on time to fully discuss medications they are prescribing, so I use apps like Stahl’s psychopharmacology (Table 1) which they themselves are using, to get the medical practitioners’ view of a particular med. Crowdsourced sites like PatientsLikeMe [58] and Iodine (Table 1 then supplement the medical view with real-world reports from patients, which is important, because clinical trials often fail to take into account patients’ viewpoints when designing research outcomes [59]. As far as medication management is concerned, setting alarms on my phone has helped me remember, for example, that I need to take my Latuda around 7 pm with dinner or it will not be fully absorbed. Some patients may want to work in a more focused way with their doctors about ensuring that they are taking their meds. For many patients, making day-to-day choices about medications reflect their own real-world priorities rather than those of their doctors [60]. This is especially true when managing serious side effects that interfere with daily functioning.

##### Distress Tolerance

Apps that might be helpful for distress tolerance really vary so much by individual. As discussed elsewhere in this paper, many patients find a music app to be critical in tolerating distressing symptoms. I find music helpful in changing my mood and motivating me to be more physical. Some people may find certain games to allow them to distract themselves long enough to get over a challenging emotional state. I belong to a Facebook group composed exclusively of people posting pictures of very fat cats. Cute animal pictures are a common method that many internet users use to self-soothe and should not be overlooked in the distress tolerance arsenal. Some may question the value of these methods when there are more evidence-based apps delivering dialectical behavior therapy and cognitive behavior therapy [61]. I cannot speak for which of these are more clinically useful than others, but when it comes to distress tolerance, I am focused less on the evidence base and more on what works for me. What works might even just be a list on a notes app of activities to try to ease distress in the moment. Furthermore, 1 trick I have found helpful for dealing with my recurrent panic attacks that I am dying is to use a pulse oximeter to remind myself that although I might feel that I am dying, if there is oxygen in my blood, my death is not imminent. I know of several other people who have gotten relief from this hack. The Apple Watch’s new electrocardiogram function (Table 1) (monitors heart rate variability, which has been linked to stress and depression [62]. Perhaps, this function can be used for reality-checking powerful feelings to tolerate them better.

##### Help With Getting Physical Activity

It is well established that exercise is beneficial to brain health [63]. I use a yoga app and an app that allows me to book exercise classes to help me get more exercise. Exercise trackers such as Fitbit and the Apple Watch allow users to passively track their exercise and encourage them to get more. In the context of encouraging patients to get more exercise, encourage them to experiment with apps that make it easier and more motivating to do so.

#### Determining What Works

How should a person suffering with mental illness go about finding good apps and evaluating their effectiveness? There is no foolproof method for doing this. App stores are full of apps. When I am searching for a new category of app to help me with a problem in my life, I look for apps that are well designed, seem easy to use, and offer appropriate push notifications. They should have plenty of reviews (I am less concerned about whether the reviews are good or bad, just that they exist) and appear to be actively supported. I prefer apps that offer trial periods and then are 1-time purchases or sold on a freemium model because I prefer to understand how a business is making its money, and apps that have income are often better-supported and a more pleasant experience to use than those that are free. Review sites on the internet (eg, Wirecutter, Table 1) offer roundups of classes of apps that can be helpful (especially for productivity apps). In my experience, the only way to truly find out if an app is effective is to download it and give it a try for a few weeks. I tried three mood-tracking apps before I settled on the one that I would use on a kind of regular basis. This is no different from trying out different medications to find one that has the right balance of symptom relief and side effects to encourage me to take it regularly.

#### Deciding How to Integrate Apps Into Clinical Sessions

Although I have just outlined several areas in which I think currently existing apps have the potential to help patients lead better lives and manage their mental illness more successfully, I want to clarify that I see apps as secondary to the core of good mental health care: personal attention from someone who cares. I do not believe that digital interventions will solve the crisis of mental health care we currently face, because in the end an app is just an app. As a result, any approach to integrating apps into mental health care must put the personal encounter front and center. A visit to a doctor or therapist should never come to resemble what so many of our medical visits now seem to be, where the doctor is increasingly focused on a computer or on data rather than on the suffering human seated in front of them. When I go to my doctor or therapist, I want their focus to be on providing empathy for my suffering and on helping me solve my problems and alleviate my symptoms as best as I can while recognizing that I suffer from an incurable chronic, debilitating disease. I do not want to be told what to do for my own good or because my doctor says so, and I do not want to spend the whole time looking at data or fiddling with devices.

Clinicians can thoughtfully inquire about how their patients are currently making use of technology to alleviate their pain and in the process of helping their patients solve problems, gently offer apps that might help solve those problems. Clinicians working with a patient on mindfulness skills might recommend that they download and try out some mindfulness apps. If recommending a new medication, a dosage change, or a lifestyle alteration that they are unsure of, clinicians can suggest tracking so that both parties use the data to make decisions *together*. If a patient is currently tracking anything, spending a couple minutes checking in about the data at the beginning or end of a session is not too time-consuming or disruptive to the main intent of the office visit, which should be about the personal interaction. If data can be emailed or provided in advance, so much the better.

##### Secure Communication With Clinicians

Being able to easily message with my therapist keeps her up-to-date on my status and provides timely assistance during crises. Most messaging methods are not particularly secure, as evidenced by reports of Facebook selling the contents of direct messages to other tech companies [64]. Owing to my experience in software, I have low expectations of privacy on internet-enabled devices. However, Signal [54] is a secure messaging app with end-to-end encryption that can be valuable for more confidential clinician-patient communications.

##### Tracking

Many clinicians see tracking as necessary and valuable to their patients’ care. Routinely tracking health can have a significant effect on health management [65], for example, moods, sleep, and other factors like medication adherence, but it is challenging in implementation. Daily tracking, although it may seem easy, is a difficult habit to start and maintain over time, and generally, people will only do it if they see some obvious benefit to it. The desire to track multiple aspects of daily life varies a lot from person to person. Recommending tracking without (1) discussing the evidence base that certain kinds of tracking may improve outcomes or (2) understanding how tracking addresses patient goals is unlikely to result in ongoing use of any tracking app. As a patient of mental health clinicians for 20 years, I do not remember a single time a clinician either took the time to go over an evidence base with me or worked with me together to find some value I could gain from tracking. And yet, I do use a simple daily mood tracker on my phone. When I made the decision to go off Latuda because I was concerned about its expense and some associated weight gain, my daily mood tracking showed that I had a very rough couple of months after that. As a result of that tracking, I decided to re-start the drug, confident that it was providing value. When my psychiatrist requested that I stop drinking for a month in exchange for agreeing that I could try stopping lithium, I saw via daily mood tracking that total abstention resulted in a more stable mood than I had experienced in the previous years during the same month, which is typically very difficult for me. I was not excited to discover the link between drinking and mood but was willing to accept it. This realization led to a significant change in my relationship with alcohol. I drink much less generally and try to abstain completely during high-risk times. I recently used my simple mood tracker to see that I had not logged a good day in 2 months. I asked my psychiatrist to re-start an antidepressant with me to improve things. Other people may have other goals for tracking (ensuring medication adherence, determining how dosing affects symptoms, tracking use of as-needed meds) or a greater tolerance for tracking more than one thing at once. Selecting mood-tracking apps that suit individual needs and motivations is critical.

#### Taking Safety and Privacy Precautions

##### Crisis Help

I am aware that the app research literature indicates that many mental health apps lack features that can be activated in a crisis [18]. However, my personal view is that I do not expect an individual app, tailored for mental health use or not, to assist me during a mental health crisis. My mobile phone itself is a simple way to get help if I need it. If a patient frequently has suicidal ideation, it is not difficult to ensure that they have the number of a crisis hotlines and other resources saved and easily accessible in their favorites. I keep my local Samaritans as a favorite in my phone, along with the numbers for my therapist and my psychopharmacologist. Many crisis hotlines accept text messages now as well as phone calls, which can lower the barrier to reaching out. I suggest that providers make sure their patients understand when and how they should reach out directly to their mental health provider versus making use of a crisis hotline.

##### Privacy and Security

As a software engineer, I am quite concerned about the privacy protections (or lack thereof) on internet-enabled devices. A recent newspaper headline stated, “Who’s making sure your mental health app is safe? No one, experts say” [66]. I know how the security and privacy internet *sausage is made*, it is full of holes, and breaches have been well documented [67]. The most realistic default assumption is that any information users provide to their devices in any app whatsoever may be subject to privacy breaches at the app level, at the operating system level, and while traveling over cellular and Wi-Fi networks. Mental health patients need to be aware that if they want to assure secrecy, they should not put personal information on their devices. I have personally made my peace with the lack of privacy in the modern world and am prepared to deal with the consequences of inadvertent breaches of my private data. Each mental health patient needs to balance the risks and benefits of their technology use.

That said, some apps can be expected to offer more privacy than others. Free services like Facebook and Google earn money off the data people provide them, including usage patterns. Email and text messaging are not secure methods of communication. If security and privacy in communications are concerns, use apps designed for more secure communications, like Signal. Some companies place a greater emphasis on privacy and security than others. Unfortunately, it can be impossible for even tech-savvy people to determine an app’s level of security. The best you can say of an app that has security and privacy front and center is that security-minded professionals have probably done some due diligence around it, that terms of service are easy to understand, and that the company’s business model is not making money from selling user data.

Privacy and security are real concerns and cannot be guaranteed. In my experience, it is helpful to accept that patients may not always feel comfortable making use of their devices. If a patient suffers from paranoia or feelings of persecution, there may be times they are more concerned about privacy and security. Feelings of paranoia are exacerbated by the real risks. For myself, I try to distinguish between my awareness that anything I say or do around internet-enabled devices might be accessed by others (which is not paranoia) and a belief that there are people out there who are persecuting me personally via my devices. (Some people may have real reasons to fear that others in their lives are tracking them via their devices, such as those in abusive relationships.) When patients cannot or do not want to use devices because of symptoms they are experiencing, it may be helpful to temporarily revert to nondigital interventions, such as tracking mood and medications on a traditional paper monthly mood chart.

##### Appropriate Use of Devices

A final consideration around the safety of using devices is that our digital devices are not an unalloyed good for anyone, including those of us struggling with mental illness. For people who experience mania, for example, they make it very easy to do a great deal of damage very quickly. If I am experiencing symptoms of mania, I can (and have) very quickly, on my phone, at any time of the day or night, spend a great deal of money with a few clicks and send multitudes of inappropriate messages. There are times when the best advice to patients may be to temporarily lock up their phones. As with a patient experiencing security worries, a patient who is causing damage to their life via their phone can revert temporarily to a predigital lifestyle. For those who have real trouble in this area, an old-style flip phone can be used during those times so that their need to be able to be in contact is balanced with limiting the damage that can be done by the combination of poor impulse control and *the world at your fingertips*.

Second, there are dark sides to our digital lives. Social apps can be used for bullying or to cause people to feel inadequate. They may contribute to consumerist and competitive values. Phones can be used addictively, sapping our focus and ability to think deeply. For those who lack concentration, excessive phone usage will make matters worse. Screen time controls and limits on use of certain apps (via built-in methods or apps like Freedom, which control usage) can be helpful to manage this problem. Sometimes low-tech methods work well. Recently I started Bullet Journaling (see Table 1). This is an entirely paper-based method of managing my thoughts and to-dos), reducing the time I spend on my devices, increasing my ability to focus, and helping me stay in the present. I still make heavy use of my devices, but more mindfully. Just as with medications, patients and doctors need to consider the costs and benefits of technology, which may change as individual symptoms wax and wane.

## Discussion

### Key Clinical Considerations and Strategies

As stated earlier, the goal of this perspective is not to generalize the experiences of 1 woman experiencing bipolar disorder to all, but to show the need for more flexible app solutions within the context of mental health treatment. On the basis of the literature and Amy’s experience, it appears that apps can be useful if they are tailored to help manage the broad psychosocial needs and symptoms that are characteristic of psychiatric problems. Amy has highlighted the value of flexible skills such as mindfulness, the use of nonmental health apps for mental health goals, anchoring app usage as an adjunct to the therapeutic relationship, the need for individuals to balance privacy considerations on personal benefits and risks, the importance of clinician explanations of evidence-based outcomes for technology use, the difficulties of overengineered apps and the value of simplicity, and the need for creative solutions such as music and games for distress tolerance. Of course, the number of apps used, the symptoms targeted, and how they will be integrated into treatment will vary greatly from person to person. Such an approach requires technology-informed clinicians and suggests that the use of apps and other digital tools should become part of clinician training.

Basing mental health app development on existing tools and platforms would recognize ways to more broadly support patients in mental health recovery and to also invite in those who have less access to potential costly and elaborate mental health apps. In addition, tools that enhance mental health goals may be better integrated into apps that are used on a regular basis. As stated by Schueller et al [4]:

...the distinction between mental health apps and health apps might be more salient to mental health researchers and practitioners than to consumers. Going a step further, it could be that the wave of the future is neither health nor mental health apps, but more mental health app features integrated into commonly used apps like our calendars, conversational agents, or messaging platforms or apps focused on health more broadly.

This would follow the concept of *nudge*, which requires less conscious thought builds on well-established, automatic responses [68]. Rather than introduce something that requires a large commitment, why not work with what people are already using (or may be interested in) and consider a nudge?

Some key questions arise. First, what would such an arrangement look like in clinical encounters? Despite the questions surrounding the long-term value of current mental health apps, reality dictates that they are a worthy topic of discussion in therapeutic interactions. Many patients are likely using mobile phones and smartwatches with apps for mental health and other health goals, so forward-thinking clinicians can find creative ways of integrating them in treatment. For example, are there ways to use apps that capitalize on healthy habits related to nutrition, mindfulness, sleep, exercise, and social support with social networks, all of which can help improve psychiatric outcomes? Clinicians may also want to review how patients can use technology to support the activities of daily living so that they can function well with their disease. Asking patients about digital tools that they use and trying to understand how they may further personal goals can be part of a therapeutic plan. Clinicians may want to discuss their availability to patients via secure messaging. Discussions about more targeted apps, for example, apps for symptom tracking, can emphasize potential advantages for a patient’s own decision-making and self-management. Finally, clinicians can play an important role in assisting their patients to adopt a responsible and balanced use of technology to avoid the possibility of social disengagement. Waiting for a mental health app that meets all the clinical criteria will only delay potential opportunities for patients to make empowered decisions by using already available technology. We need to maximize current tools until that day (hopefully) arrives.

There has also been increasing interest in delivering interventions that address symptoms that cross diagnostic boundaries between anxiety, depression, and other psychiatric disorders [18,69,70]. Interventions such as Intellicare [71] capitalize on this notion. A recent consumer evaluation of apps targeting bipolar disorder found that users requested features such as multiple category–tracking (sleep, medication, diet, mood triggers, exercise, anxiety, and substance use), ability to track fluctuations throughout the day, access to a Web-based community, and data-sharing abilities with clinicians [9]. Rather than funneling patients into single disorder apps, there may be opportunities for mental health providers to suggest combinations of apps that best meet individual patient needs [72]. Such a *toolbox* approach may better address the problems that patients face, reduce stigma, and ultimately encourage app adoption. This approach can also be implemented today by any clinician, using features and apps that are already available.

Second, if mental health apps are to be considered as part of an intervention, how can the issues of quality and training be addressed? The issue of quality cannot be separated from the need for clinician education, as the National Institute of Mental Health has stressed the need for the development of new technology-training models for clinician and task shifting through technology apps delivered by trained paraprofessionals [73]. A part of this training should be devoted to the best ways to educate patients about the value of digital health tools and how clinicians can utilize these tools in treatment. There are app standards developed by academic groups (PsyberGuide [74]), private companies (AppScript [75]), professional organizations (American Psychiatric Association App Evaluation Model [76]), and governmental organizations (United Kingdom–based National Health Service Apps Library [77]). These groups have attempted to establish more rigorous evaluation standards based on the ease of use, clinical value, rigorous evidence-base outcomes, and a regard for patient safety and privacy. Torous et al [72] have developed an acronym called ASPECTS (Actionable, Secure, Professional, Evidence-based, Customizable, TranSparent) to describe ideal characteristics of health apps and to help patients. These resources and criteria can be used to help patients and clinicians identify the latest health technology, for example, tracking patient functioning between office visits.

Finally, how can the approach described in this paper be leveraged to address the needs of those who are not ready or able to access treatment or who lack an understanding of how to use apps to enhance their mental health? Those who might benefit the most from using health apps, that is, poor health or inadequate physical activity, are least likely to download and utilize digital health tools [17]. Such a finding makes it more imperative to reach outside of a mental health framework to identify ways in which mental health interventions (emotional management, countering negative cognitions, coping with symptoms such as fatigue and pain, and improving sleep) can be integrated into daily scheduling, goal setting, and social media apps. Mobile phone penetration is high across all strata, as almost every developing country has reached a rate of 90% ownership [78]. In addition, mobile phone users and app users are virtually synonymous—only 1 in 10 mobile phone users does not use apps [79]. By eliminating the barrier of labeling an app for *mental health* (or even health), there may be opportunities to invite new audiences to interventions that can improve their mental health in the context of lifestyle management.

### Conclusions

We believe that the application of mental health apps and other technologies in psychiatric treatment has not sufficiently accounted for the nuanced expectations, experiences, and challenges that patients face when trying to make practical use of technology. Amy’s experience shows how 1 woman has developed a personalized and creative strategy with mental health apps to cope with bipolar disorder. We believe that the key to effective deployment of apps in psychiatric treatment rests on the flexible use of apps rather than sole reliance on condition-specific (*mental health*) apps. Rather, the use of technology should address the broad range of lifestyle challenges presented by bipolar and other mental disorders. On the basis of the existing literature and examples such as Amy’s lived experience, there are potential avenues for further hypothesis testing and program development with such tailored strategies.
